# Month-wise variation and prediction of bulk tank somatic cell count in Brazilian dairy herds and its impact on payment based on milk quality

**DOI:** 10.1186/s13620-017-0103-z

**Published:** 2017-08-15

**Authors:** Marcos Busanello, Larissa Nazareth de Freitas, João Pedro Pereira Winckler, Hiron Pereira Farias, Carlos Tadeu dos Santos Dias, Laerte Dagher Cassoli, Paulo Fernando Machado

**Affiliations:** 1 Department of Animal Science, College of Agriculture, “Luiz de Queiroz”/University of São Paulo - ESALQ/USP, Campus Piracicaba, São Paulo, 13418-900 Brazil; 2 Department of Exact Sciences, College of Agriculture, “Luiz de Queiroz”/University of São Paulo - ESALQ/USP, Campus Piracicaba, São Paulo, 13418-900 Brazil

**Keywords:** Dairy science, Mastitis, Payment by milk quality, Management, Financial losses, Bonuses and penalties, Time series analysis

## Abstract

**Background:**

Payment programs based on milk quality (PPBMQ) are used in several countries around the world as an incentive to improve milk quality. One of the principal milk parameters used in such programs is the bulk tank somatic cell count (BTSCC). In this study, using data from an average of 37,000 farms per month in Brazil where milk was analyzed, BTSCC data were divided into different payment classes based on milk quality. Then, descriptive and graphical analyses were performed. The probability of a change to a worse payment class was calculated, future BTSCC values were predicted using time series models, and financial losses due to the failure to reach the maximum bonus for the payment based on milk quality were simulated.

**Results:**

In Brazil, the mean BTSCC has remained high in recent years, without a tendency to improve. The probability of changing to a worse payment class was strongly affected by both the BTSCC average and BTSCC standard deviation for classes 1 and 2 (1000–200,000 and 201,000–400,000 cells/mL, respectively) and only by the BTSCC average for classes 3 and 4 (401,000–500,000 and 501,000–800,000 cells/mL, respectively). The time series models indicated that at some point in the year, farms would not remain in their current class and would accrue financial losses due to payments based on milk quality.

**Conclusion:**

The BTSCC for Brazilian dairy farms has not recently improved. The probability of a class change to a worse class is a metric that can aid in decision-making and stimulate farmers to improve milk quality. A time series model can be used to predict the future value of the BTSCC, making it possible to estimate financial losses and to show, moreover, that financial losses occur in all classes of the PPBMQ because the farmers do not remain in the best payment class in all months.

## Background

The purpose of payment programs based on milk quality (PPBMQ) is to improve milk quality via a monetary incentive that is paid to the farmer per liter of milk. To do this, the milk buyers establish ranges or classes of payment based on milk quality according to various components and indicators, such as milk constituents (fat and protein) and the microbiological and sanitary quality of the product (somatic cell count - SCC and total bacterial count - TBC) [1, 2].

The classes and amount paid may vary between milk buyers, and the farmers are paid for the class to which their milk was assigned in a particular month. The PPBMQs are usually based on a bonus system, a penalty system or both [3], and some studies indicate that PPBMQs based on penalty systems are the most effective to stimulate the farmers to improve the bulk tank SCC (BTSCC) [4, 5]. One of the indicators most often used in these programs is the SCC, which is based on the geometric mean monthly of BTSCC for the farm. Considering that the SCC has a negative correlation with lactose and casein, a high SCC value consequently leads to a reduction in the dairy products yield [6]. Therefore, this quality indicator is one of the most relevant for milk buyers and dairy companies.

Payment programs based on milk quality have only recently been studied. Some of those studies aimed to determine whether farmer participation in PPBMQs was associated with an improvement in milk composition [1, 7, 8], while others aimed to evaluate the improvement of the microbiological and sanitary quality of the milk (SCC and TBC) mainly with respect to mastitis management in the farms [2–5, 9]. Moreover, some studies have related financial questions to PPBMQs, but the approaches vary among the studies. Hand et al. [10] investigated whether access to cow-level SCC data for farmers could be associated with a decrease in the risk of penalties based on the BTSCC (*n* = 4084 herds); Banga et al. [11] studied the economic value of the BTSCC for Holstein and Jersey herds, indicating that it varies by breed, payment scheme and production system (*n* = 392 herds); Sadeghi-Sefidmazgi and Amer [12] studied the economic benefits originating from the reduction of BTSCC (*n* = 25 herds); and Teixeira Júnior et al. [13] used simulations to study the effect of PPBMQs on a dairy farm profitability. Still, there have been few studies addressing financial losses originating from PPBMQs.

Bulk tank somatic cell count can vary from month to month [14], and even farmers who are receiving the maximum bonus for milk with a low BTSCC value for a given milk buyer may not receive this maximum bonus in subsequent months. Thus, normally, farmers cannot achieve the PPBMQ class that would allow the maximum bonus during all months of the year because they shift to worse classes in the PPBMQ (with smaller bonuses/more penalties). Often, farmers do not realize that failing to earn the maximum bonus is a financial loss, instead considering losses to be only penalties imposed [3, 4].

Thus, the probability that the payment class for a farm will change to a worse class, considering the variability of the BTSCC between months [15], is a metric that can help farmers make decisions in terms of the improvement of the BTSCC. Moreover, other methods, such as time series analysis, also can be used to predict when the change to a worse payment class might occur. Such information would enable the farmer to reduce the BTSCC in time to avoid the cessation of a bonus or the imposition of a penalty.

Statistical models known as time series are generally employed to predict the future value of a given variable based on past values. This method has also been used in a study to predict future values for BTSCC in Ontario, Canada [16]. This type of model would enable the farmer to predict future quality levels and to act in time to reduce the BTSCC and to not exceed the maximum limit of the current PPBMQ class. In addition, an expected value can be used to estimate the financial losses related to PPBMQ for each BTSCC class. Moreover, time series models consider the internal structure of the data, such as autocorrelation between observations and effects of tendency (of increase or decrease of BTSCC over time) and seasonality [17]. Since such models allow the prediction of future results of BTSCC, a farmer can thus take action before a negative result occurs, for example, dry off some cows with high SCC values or discard the milk of such cows to reduce the BTSCC value in the next month.

Therefore, the objectives of this study were as follows: i) to verify the probability of class change to a worse class in a PPBMQ based on the mean and estimated standard deviation of the BTSCC of a farm, ii) to predict the future values of BTSCC based on the mean BTSCC of farms in each payment class of the PPBMQ, and iii) to simulate the financial losses resulting from a failure to accrue the maximum possible PPBMQ bonus.

## Methods

### Study design and database characterization

The STROBE statement [18] was used as a guideline for reporting the results of this study. This research was designed as a longitudinal retrospective study and used BTSCC data from Brazilian dairy farms from January 2011 to February 2016. The farms sold the milk to milk buyers that sent samples of the bulk tank milk for SCC analysis to the laboratory of the Clínica do Leite, at College of Agriculture “Luiz de Queiroz”/University of São Paulo (ESALQ / USP).

From each farm, a total of 3–5 milk samples were collected monthly by buyers’ employees. In this way, the number of samples per month per farm was not the same to calculating BTSCC geometric mean for a given month for each farm. Thus, the value of BTSCC geometric mean for each farm in each month was considered as being a representation of the BTSCC for the 30 days of the month. The milk came from 421 dairy companies located mainly in the Southeast (86%) and the South (4.5%), with the rest of the milk coming from other regions of the country (9.5%). On average, milk from 36,929 farms was analyzed for each month during the study period. However, as the data were collected by dairy companies and information about the farms (average herd size, calving pattern, average milk production, production system, etc.) is not required by these companies, only BTSCC information was available in the database.

### Payment based on milk quality according to the BTSCC

Since payment based on milk quality is applied in different ways by the milk buyers, it was necessary to create a reference list for this study with milk quality classes and the amounts of bonuses and penalties in *R$* (Brazilian currency). Bonuses and penalties in milk-equivalents (a parameter that represents a financial value equivalent to a liter of milk, which has also been used by Madalena [19] and Martins et al. [20]) were also calculated so that the values could be extrapolated to other countries. For this purpose, an average milk price of *R$* 1.34 per liter [*€* 0.36] was used, corresponding to the price during the months between January and November 2016 (Center for Advanced Studies in Applied Economics - CEPEA-ESALQ/USP).

Based on the existing tables of 13 milk buyers in Brazil that used PPBMQs with their suppliers, it was possible to elaborate a base table to be used with the bonus and penalty system (Table 1) [3]. This allowed the creation of five payment classes for milk quality according to the BTSCC: Class 1 (1000–200,000 cells/mL), Class 2 (201,000–400,000 cells/mL), Class 3 (401,000–500,000 cells/mL), Class 4 (501,000–800,000 cells/mL), and Class 5 (> 800,000 cells/mL). The bonus/penalty amounts per liter were as follows for classes 1, 2, 3, 4 and 5, respectively: in *R$*, +0.04, +0.02, 0, −0.01, and −0.02; and in milk-equivalents, +0.030, +0.015, 0.000, −0.007, and −0.015 l.

**Table 1:** Payment by bulk tank somatic cell count (BTSCC) for each class and the respective adittion in the payment per liter of milk in R$ and in milk-equivalents^a^

Class	BTSCC _×_ 1000 cells/mL	Payment adittion (R$)/liters	Milk-equivalents (liters)
Class 1	1–200	0.040	0.030
Class 2	201–400	0.020	0.015
Class 3	401–500	0.000	0.000
Class 4	501–800	−0.010	−0.007
Class 5	> 800	−0.020	−0.015

^a^This table is based on the payment programs based on milk quality of 13 milk buyers in Brazil

### Probability of changing to a worse class

The objective of this analysis was to estimate whether the chance that a farm will be shifted to a worse class of the PPBMQ is affected by the mean and variation of BTSCC. This analysis was applied only to classes 1, 2, 3 and 4 because there are no worse classes than class 5, so farms in class 5 cannot be shifted to a worse class. For the calculation of the probability of a change to a worse class, the monthly geometric means of BTSCC from the farms were used. For example, when BTSCC was measured 5 times within 1 month on one farm, the geometric mean for that farm was calculated in that month. In addition to the monthly average, the monthly variability of the BTSCC was an important consideration in calculating the probability of class change. However, although it was possible to calculate standard deviation within a given month for the same farm, we judged that such a measure was not an appropriate one to study the variation between months. For this reason, BTSCC variation was estimated based on two consecutive results of BTSCC average (considering BTSCC averages as moving averages, i.e., measurements made over time), making it necessary to estimate a standard deviation for the BTSCC average (it is described in some texts about statistical process control charts – [21, 22]). Thus, the difference between the values for two consecutive months was calculated for the same farm to obtain the moving range between months [15]. Then, the value of the moving range was divided by the tabulated value *d*_*2*_ of 1.128 [23] in order to estimate the standard deviation of the moving average as reported by Lukas et al. [15]. Finally, the estimated standard deviation was associated to the average BTSCC of the current month. For example, for one farm, the BTSCC average was 250,000 cells/mL in January 2015 and 220,000 cells/mL in February 2015; the difference between months was 30,000 cells/mL (moving range); dividing 30,000 cells/mL by 1.128 (*d*_*2*_) provides the estimated standard deviation of 26,595 cells/mL; so, for this farm in February 2015, the BTSCC average and the BTSCC variation will be 220,000 and 26,595 cells/mL, respectively.

Subsequently, by verifying whether each farm had or had not exceeded the maximum limit of its current class in the next month, the farms were classified according to the change in class in the following month. Thus, each farm was allocated to a payment class (1–5) based on the geometric mean for BTSCC recorded for a given month. The chances of a farm remaining in the same class or moving to a better payment class were not calculated because it is considered that the chance of a loss (passing to a worse class) is more effective for motivating the farmers to improve milk quality (loss aversion [3, 5]). Each month, based on the actual BTSCC average and BTSCC standard deviation for that month, a mean and a variation category were assigned to each herd [15]. For each of the payment classes (1, 2, 3 and 4), categories were assigned for BTSCC standard deviation (0 to 200,000 cells/mL, step 50,000 cells/mL, and >200,000 cells/mL for all classes) and for BTSCC average (0 to 200,000 cells/mL, step 50,000 cells/mL, for class 1; >200,000–400,000 cells/mL, step 50,000 cells/mL, for class 2; >400,000–500,000 cells/mL, step 50,000 cells/mL, for class 3; and >500,000–800,000 cells/mL, step 50,000 cells/mL, for class 4), forming quadrants. In this way, the number of quadrants for each class was 5 to BTSCC standard deviation and 4, 4, 2 and 6 to BTSCC average for classes 1, 2, 3, and 4, respectively. Finally, after combining quadrants of BTSCC standard deviation and BTSCC average, we had a total of 20, 20, 10 and 30 quadrants in classes 1, 2, 3, and 4, respectively.

A farm was classified in the appropriate quadrant according to the monthly geometric mean and the estimated standard deviation. For example, for a given month, a farm with BTSCC average and standard deviation of 140,000 and 15,000, respectively, was classified in class 1 and in the quadrant for the BTSCC average of 100,000–150,000 cells/mL and BTSCC standard deviation of ≤50,000 cells/mL. Then, the class change probability (%) for each quadrant was obtained, calculating the proportion of farms that passed to a worse payment class in the following month. In the calculation, the chance of class change for a farm in a month, for example, to pass of class 1 to class 2 or to pass of class 1 to class 3 (or other higher class), were treated in the same way.

A multiple linear regression analysis was performed to verify whether the probability of passing to a worse class was related to an increase in the mean and standard deviation of the BTSCC. For this, we had 3 data points per quadrant: one change probability to a worse class, one median of all BTSCC geometric means of the farms and one median of all estimated BTSCC standard deviation of the farms. Values of the class change probability (%) of the quadrants were used as dependent variable and the medians obtained from the BTSCC average values and from the BTSCC standard deviations for the group of farms were used as independent variables in the statistical model. A single farm could participate with more than one measure on the calculation of class change in one quadrant. For example, if one farm remained in the same quadrant for 5 months, its 5 measures were used in the calculation of class change probability and on the median calculation.

First for the statistical analysis, all the farms in the original database were considered, where the proportion of farms that changed to a worse PPBMQ class in each quadrant was calculated for each of the classes 1, 2, 3 and 4 (class 5 was not used in this analysis because there is no worse class to pass into). This analysis was performed using SAS PROC FREQ over the class change information (1 = yes; 0 = no), obtaining the proportion of farms that passed to a worse class in the following month. Subsequently, SAS PROC REG was used for the multiple linear regression analysis and comprised the following Eq. (1):

1 $$ \gamma ={\beta}_0+{x}_1{\beta}_1+{x}_2{\beta}_2+\varepsilon . $$

Where.

*γ*_:_ probability of changing to a worse class,

*β*_0_: intercept,

*x*_1_ *β*_1_: effect of the BTSCC geometric mean of the farms,

*x*_2_ *β*_2_: effect of the BTSCC standard deviation of the farms,

*ε*_:_ random error.

For this model, the assumptions of homoscedasticity, normality and linearity were evaluated by a graphical analysis of the standardized residues against the adjusted predicted values, the quantile-quantile of the standardized residues (normal probability plot) and the standardized residues against the predictor variables, respectively [24]. The assumption of multicollinearity between the independent variables was tested by checking the variance inflation factor (VIF), where VIF = 1 indicates that variables are uncorrelated, a VIF between 1 and 5 indicates moderate correlation, and a VIF between 5 and 10 indicates a high degree of correlation [25]. The VIF assesses the degree to which an independent variable can be predicted by the other independent variables in the model. However, for all assumptions to be met, it was necessary to use a log_10_ transformation of the dependent variable (probability of passing to a worse class) in classes 2, 3 and 4. Class 1 met all the assumptions and therefore did not require transformation. In sequence, for each one of above-mentioned classes, one multiple linear regression was performed. The dataset had unequally spaced repeated measurements because farms frequently changed their current payment class, and also because results of many farms were not sent frequently, resulting in long periods without observations of such farms. These were the main reasons why we chose not to use a repeated measures approach in the analysis. Therefore, farm effect was not considerate in this statistical analysis. The analysis and the probability calculations were performed with SAS software, version 9.3 SAS/2012. Differences were considered significant when *p* < 0.05 (5%). The calculation of the BTSCC geometric means were done with R software (www.r-project.org) with RStudio (RStudio, 2012; Version 0.99.902; RStudio, Boston, Massachusetts, US) using the packages *psych* [26].

### BTSCC prediction by time series model

Unlike in the above-mentioned analysis, all 5 classes were used for this analysis. The objective of this analysis was to predict the BTSCC for each class from a time series that originated from the group of farms present in that class. Only farms with 60 observations were selected for the time series modeling, i.e., 60 months of BTSCC analysis were obtained in the period from January 2011 to December 2015. Therefore, samples from the farms must have been analyzed every month for 5-years, for a total of 1013 farm samples. Subsequently, a general BTSCC geometric means for the 5-year period was calculated for each of the selected farms. Thus, the farms were classified within the classes of the elaborated PPBMQ based on these calculated BTSCC means (Table 1). In sequence, each farm (allocated into one of the classes 1–5) also contributed to the dataset with a geometric mean per month for the 5-year period.

In this manner, 110, 429, 170, 236 and 68 farms were assigned to classes 1, 2, 3, 4 and 5, respectively. To generate a time series for use in the modeling for each class, the monthly BTSCC geometric means were calculated for all the months (January 2011 to December 2015) considering the monthly BTSCC values of the farms grouped in each class. Several models were tested for each class, and a seasonal effect was identified in all classes. Thus, the most adequate model for the correct modeling of the time series was a seasonal autoregressive integrated moving average model (SARIMA). This model accounted for the stochastic seasonality of the data, in addition to considering the collection order of the data and the dependence between neighboring observations in the model (the greater the proximity of the observations, the greater is the dependence between them) [17]. When a period s = 12 occurred, as in this study, a SARIMA model of the order (*p*,*d*,*q*) × (*P*,*D*,*Q*)_12_ is given by Morettin and Toloi [27] as Eq. (2):

2 $$ \varphi (X)\varPhi \left({X}^{12}\right){\Delta}^d{\Delta}_{12}^D{Z}_t=\theta (X)\varTheta (X){a}_t. $$

wher e.

*φ*(*X*) is the non-seasonal autoregressive operator (AR) of order *p*,

*θ*(*X*) is the non-seasonal moving-averages operator (MA) of order *q*,

*Φ*(*X*) is the AR-seasonal operator of order *P*,

*Θ*(*X*) is the MA-seasonal operator of order *Q*,

∆^*d*^ is the operator difference,

${\Delta}_{12}^D$ is the operator seasonal difference and,

*a*_*t*_ is white noise (errors are uncorrelated).

Model 1 was designated as a seasonal multiplicative ARIMA of order (*p*, *d*, *q*) × (*P*, *D*, *Q*)_12_ and represented as SARIMA(*p*, *d*, *q*) × (*P*, *D*, *Q*)_12_. A SARIMA model indicates that *d* simple differences and *D* seasonal differences of the data series *Z*_*t*_ should be used, considering that the analysis process develops around a constant average and has a constant variability. It is necessary to examine differences between data points when we have seasonality, because seasonality can cause nonstationary series considering that the average values at some particular moment within the seasonal period (years, in this study) may differ from the average values at other moments. Stationary series is one assumption of time series analysis, meaning that the time series keeps a constant mean over time. A balance is demonstrated within the time series, even though many time series present with some type of nonstationarity, for example, tendency and seasonality. Tendency and seasonality can be detected and modeled within the model to provide a stationary series. In our case, the analysis process could be identified as weakly stationary, which is a necessary condition for adjusting a time series model [28].

The modeling was continued with an iterative cycle of specification, identification, estimation and diagnosis of the adjusted model. The SARIMA model class was considered during the specification process, with the aid of the graphs of the time series and a periodogram. The autocorrelation functions (ACF) and partial autocorrelation functions (PACF) of the residuals were used to identify the orders *p*, *q*, *P* and *D*, and the models were selected by evaluating the Akaike information criterion (AIC) and Bayesian information criterion (BIC). To estimate the parameters of the proposed models, we used the maximum likelihood estimators, and to verify the quality of the adjustment, we observed the ACF and PACF graphs of the residuals and the Ljung-Box test, for which the hypothesis is that the errors are uncorrelated [29]. The BTSCC classes, the selected models and the respective Ljung-Box values are presented in Table 2.

**Table 2:** Time series models used for each payment program based on milk quality (PPBMQ) class based on milk quality and the respective value of the Ljung-Box test

Class	Model	*p*-value (Ljung-Box test)
Class 1^1^	SARIMA(2,1,0)(2,1,0)_12_	0.41^NS^
Class 2^1^	SARIMA(2,1,0)(1,1,0)_12_	0.51^NS^
Class 3^1^	SARIMA(1,1,0)(1,1,0)_12_	0.31^NS^
Class 4^1^	SARIMA(0,1,1)(0,1,2)_12_	0.63^NS^
Class 5^1^	SARIMA(1,1,1)(0,1,1)_12_	0.81^NS^

^NS^– non-significant; ^1^Class 1–1000 to 200,000 cells/mL, Class 2–201,000 to 400,000 cells/mL, Class 3–401,000 to 500,000 cells/mL, Class 4–501,000 to 800,000 cells/mL, Class 5 - > 800,000 cells/mL

The calculation of the BTSCC geometric means and the time series modeling, the assumptions for the modeling and the model selection were tested with R software (www.r-project.org) with RStudio (RStudio, 2012; Version 0.99.902; RStudio, Boston, Massachusetts, US) using the packages *psych* [26] for the geometric means, *forecast* [30, 31] and *tseries* [32] for the time series model, and *ggplot2* [33] for the graphical analyses.

### Simulation of the financial loss related to the payment based on milk quality

For this simulation, all 5 classes were used in the analysis. After obtaining the predicted values (future values of BTSCC median to the year 2016 as obtained through time series analysis) for each BTSCC class, the financial loss due to the non-attainment of the maximum bonus for the milk quality payment was calculated, considering the PPBMQ reference table (Table 1). To accomplish this, the values of losses in milk-equivalents per liter of milk per month were added, and the average annual loss for each the BTSCC class was calculated. The monthly average of the financial loss from the PPBMQ per liter of milk was used to calculate the daily percentage (%) of liters that would correspond to the loss value. The value of daily production of milk was hypothesized for a farm with production of 1000 l per day, but the values of “Liters per day (%)” are independent of the production level considering that they were calculated in percent. This calculation used a profit margin of 15% and a per-liter milk price of R$ 1.34 in Eq. (3):

3 $$ Liters\  per\  day\ \left(\%\right)=\frac{\left({DP}^{\ast } AFLC\right)}{\left({PM}^{\ast } MP\right)}/ DP $$

where.

*DP*: daily production in liters of milk,

*AFLC*: average financial loss of the BTSCC class per liter of milk related to the non-attainment of the maximum bonus,

*PM*: profit margin (assumed to be 15%) and,

*MP*: milk price (assumed to be R$ 1.34 per liter = 1 milk-equivalent).

## Results

### BTSCC over the course of the study

**Fig. 1:**
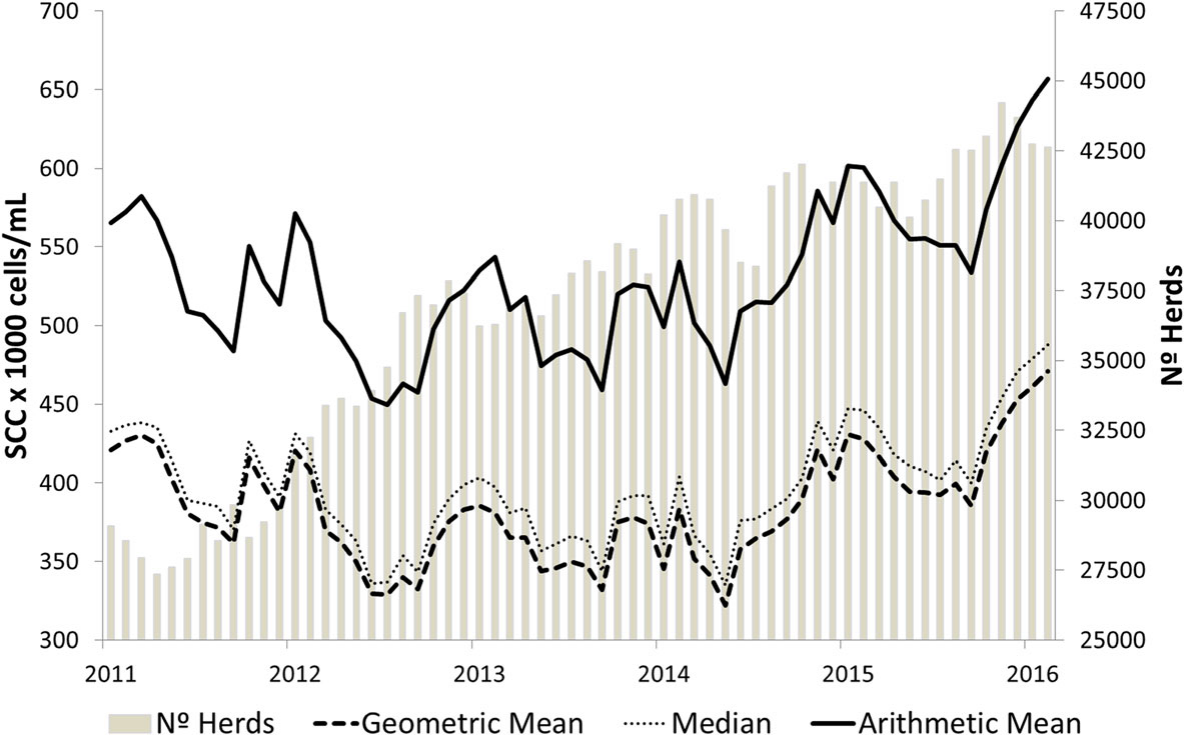
Behavior of bulk tank somatic cell count (BTSCC) and number of dairy farms in Brazil during the months of January/2011 to February/2016. For BTSCC, geometric mean, arithmetic mean and median are presented, whereas for the number of dairy farms, the arithmetic mean is presented

Considering the entire database, based on one BTSCC geometric mean for all the farms with data in any given month, the BTSCC arithmetic mean, geometric mean and median on the period (January 2011 to February 2016) were calculated, as was the number of Brazilian farms for which milk was analyzed (Fig. 1). The arithmetic mean of the BTSCC of the period was 530,000 cells/mL with a 95% confidence interval (CI) = 519,000–542,000 cells/mL, while the geometric mean was 382,000 cells/mL, and the median (a robust measure) was 392,000 cells/ml. The average number of farms per month for which the BTSCC was determined was 36,929 (95% CI = 35,691–38,168 farms).

**Fig. 2:**
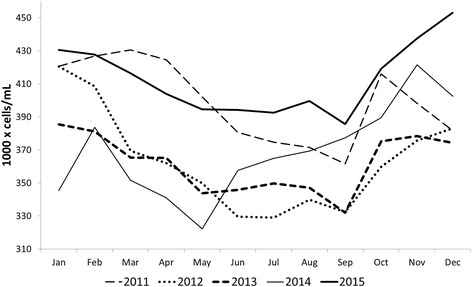
Behavior of bulk tank somatic cell count geometric mean (BTSCC) in the months of the years 2011 to 2015 in Brazil

On the other hand, there was an increase in the BTSCC geometric mean in later years (Fig. 2), with values of 399,000, 362,000, 362,000, 368,000 and 412,000 cells /mL for the years 2011, 2012, 2013, 2014 and 2015, respectively. In 2011, the average number of farms surveyed monthly was 28,635 (95% CI = 28,176–29,094 farms); this measure increased in the following years to 34,937 (95% CI = 33,688–36,186 farms), 37,618 (95% CI = 37,038–38,198 farms), 40,533 (95% CI = 39,877–41,190 farms) and 41,962 (95% CI = 41,242–42,683 farms) in 2012, 2013, 2014 and 2015, respectively.

### Probability of class change based on payment based on milk quality

The means of the BTSCC geometric means and means of standard deviations for the farms in each class were class 1–132,000 ± 118,000 cells/mL, class 2–297,000 ± 124,000 cells/mL, class 3–448,000 ± 148,000 cells/mL, class 4–629,000 ± 192,000 cells/mL, and class 5–1,340,000 ± 497,000 cells/mL (class 5 was not used for this analysis).

To determine whether the probability of changing to a worse class was associated with the BTSCC means and standard deviations, a multiple linear regression analysis was performed for each of the classes (i.e., 1, 2, 3 and 4). The analysis showed that for classes 1 and 2, the BTSCC mean and standard deviation had a significant positive association (*p* < 0.05) with the class change probability (Table 3), i.e., a higher BTSCC mean and standard deviation of a farm indicated a greater probability of class change. For classes 3 and 4, as expected based on the observed probabilities, a significant effect occurred only for the BTSCC mean (*p* < 0.05), i.e., the standard deviation for these BTSCC classes was not a significant variable within the statistical model and did not affect the probability of class change. However, a considerable number of farms frequently changed their current payment class. Considering this, BTSCC standard deviation was maintained in the models for the classes 3 and 4, because the farmers could get used to using such a measure, which will facilitate the calculation to them when they reach to a class (1 and 2) where this measure was significant and need to be used.

**Table 3:** Multiple linear regressions analyses by payment class based on milk quality according to bulk tank somatic cell count (BTSCC) means and standard deviations (in this analysis only class 1, 2, 3 and 4 were used)

Class	Variable	Estimated parameter	Std. Error^3^	*p*-value*	R^2^-Adjusted
Class 1^1^	Intercept	11.87	2.98	0.001	0.88
	BTSCC mean	1.83^−4^	2.17^−5^	<0.001	
	Standard deviation	8.39^−5^	1.00^−5^	<0.001	
Class 2^1,2^	Intercept	0.66	0.07	<0.001	0.88
	BTSCC mean	2.27^−6^	2.33^−7^	<0.001	
	Standard deviation	9.69^−7^	1.32^−7^	<0.001	
Class 3^1,2^	Intercept	0.52	0.20	0.324	0.74
	BTSCC mean	2.32^−6^	4.49^−7^	0.001	
	Standard deviation	−1.61^−7^	1.42^−7^	0.292	
Class 4^1,2^	Intercept	−0.05	0.07	0.456	0.93
	BTSCC mean	2.07^−6^	1.04^−7^	<0.001	
	Standard deviation	7.37^−8^	9.03^−8^	0.421	

*Statistically significant difference considered *p* < 0.05; ^1^Class 1–1000 to 200,000 cells/mL, Class 2–201,000 to 400,000 cells/mL, Class 3–401,000 to 500,000 cells/mL, Class 4–501,000 to 800,000 cells/mL, Class 5 - > 800,000 cells/mL; ^2^dependent variable was transformed to log_10_; ^3^Standard error

The equations below were obtained by using the multiple linear regression models and can be used to calculate the probability of class change:

Class 1:

*PROB* = 11.87 + (1.83^−4^ * *MEAN* ) + (8.39^−5^ * *STD*)_,_(4)

Class 2:

*PROB* = 0.66 + (2.27^−6^ * *MEAN* ) + (9.69^−7^ * *STD*),(5)

Class 3:

*PROB* = 0.52 + (2.32^−6^ * *MEAN*) + (−1.61^−7^ * *STD*),(6)

Class 4:

*PROB* =  − 0.05 + (2.07^−6^ * *MEAN* ) + (7.37^−8^ * *STD*),(7)

where.

*PROB*: probability of passing to a worse class,

*MEAN*: BTSCC monthly mean of the farm and,

*STD*: BTSCC estimated standard deviation of the farm.

The variable response was not transformed in class 1, but for classes 2, 3 and 4, log_10_ transformation was necessary. Therefore, the equation should be used as a power of 10 to obtain the values for the probability of passing to a worse class already in percentage form (%).

### Prediction of the BTSCC

**Fig. 3:**
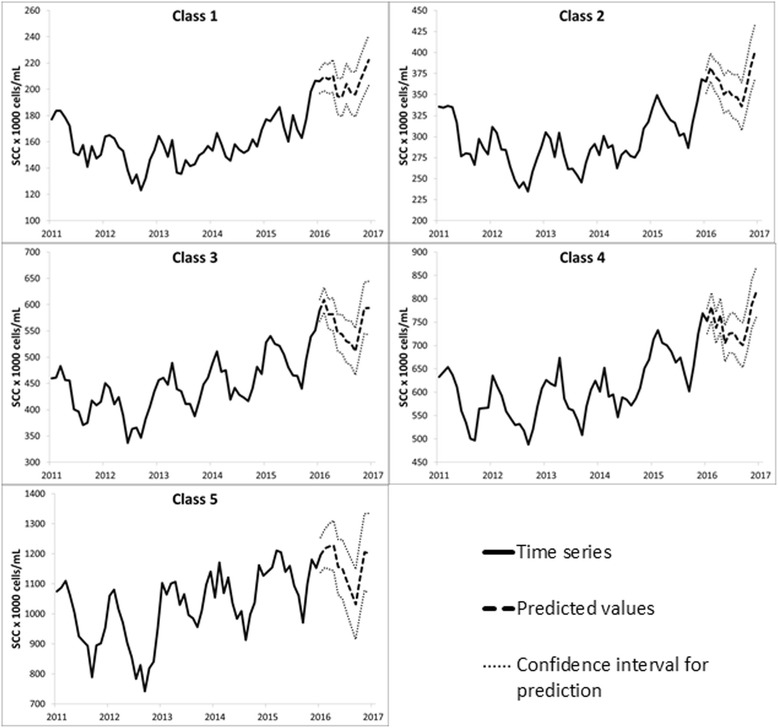
Time series analysis with predicted values and confidence interval for prediction of bulk tank somatic cell count (BTSCC) for each payment program based on milk quality (PPBMQ) class. In each graph, we present the BTSCC geometric mean for farms in each month in each PPBMQ class for 2011 to 2015, and their respective predicted values and the confidence interval for the prediction for 2016. The classes were: Class 1–1000 to 200,000 cells/mL, Class 2–201,000 to 400,000 cells/mL, Class 3–401,000 to 500,000 cells/mL, Class 4–501,000 to 800,000 cells/mL, Class 5 - > 800,000 cells/mL)

All five classes were used in this analysis. To predict future values of BTSCC, 1013 farms were used and allocated to the respective PPBMQ classes according to their geometric means in the study period. Then, a time series was obtained from the observations of the farms in the each month for each class. For example, to obtain the BTSCC geometric mean point for January 2013 for class 1, the observations of all the farms in class 1 for this month were used in the calculation. Respectively, 110, 429, 170, 236 and 68 farms were assigned to classes 1, 2, 3, 4 and 5. The time series graphs were created with the monthly BTSCC geometric means for the groups of farms from each class (Fig. 3).

In the graphs, it is possible to identify the time series for each class and the predicted values within the confidence interval for prediction (Fig. 3 and Table 4). The time series modeling also indicated a seasonal effect, with a 12-month interval, and a trend for all classes. The trend is present in the graphs for all classes, for which an increase in the average annual BTSCC from the year 2012 was shown (Fig. 3). Seasonality was a characteristic of our BTSCC data: in cool months BTSCC normally decreases and in hot months it normally increases, as demonstrated with our results. In general, the predictions of the time series models were efficient, with fairly safe and acceptable forecast errors (Table 4).

**Table 4:** Future values of bulk tank somatic cell count (BTSCC) ± forecast error obtained from time series modeling per payment program based on milk quality (PPBMQ) class for the months of 2016^a^

	BTSCC predicted value ± standard error × 1000 cells/mL				
Month	Class 1^1^	Class 2^1^	Class 3^1^	Class 4^1^	Class 5^1^
January/2016	206 ± 9	366 ± 14	589 ± 21	753 ± 27	1196 ± 58
February/2016	210 ± 11	382 ± 16	609 ± 24	783 ± 30	1218 ± 64
March/2016	208 ± 11	372 ± 18	582 ± 28	738 ± 33	1224 ± 76
April/2016	210 ± 12	366 ± 20	582 ± 31	765 ± 36	1227 ± 83
May/2016	195 ± 14	350 ± 22	546 ± 34	704 ± 39	1156 ± 91
June/2016	194 ± 14	355 ± 24	544 ± 37	725 ± 41	1149 ± 98
July/2016	204 ± 15	349 ± 26	530 ± 40	727 ± 43	1108 ± 105
August/2016	197 ± 16	347 ± 27	527 ± 42	710 ± 46	1069 ± 111
September/2016	196 ± 17	336 ± 28	511 ± 45	701 ± 48	1032 ± 117
October/2016	206 ± 18	357 ± 30	552 ± 47	737 ± 50	1122 ± 123
November/2016	214 ± 18	382 ± 31	593 ± 49	788 ± 52	1205 ± 128
December/2016	222 ± 19	401 ± 32	594 ± 51	814 ± 54	1202 ± 133

^a^The classes were based on the payment programs based on milk quality of 13 milk buyers in Brazil; ^1^Class 1–1000 to 200,000 cells/mL, Class 2–201,000 to 400,000 cells/mL, Class 3–401,000 to 500,000 cells/mL, Class 4–501,000 to 800,000 cells/mL, Class 5 - > 800,000 cells/mL

### Financial losses related to payment based on milk quality

To calculate the financial losses related to the payment based on milk quality, a monthly loss table was created based on the expected BTSCC values from the time series models (Table 5). The average annual losses per liter of milk were calculated for each class from this analysis as a hypothetical model for a particular situation. It was possible to calculate the number of additional liters of milk that needed to be produced per day to equal losses resulting from failure to reach the maximum bonus by considering the value of a liter of milk as R$ 1.34 (1 milk-equivalent), a profit margin of 15%, and the average annual financial loss due to the PPBMQ based on the BTSCC and the daily milk production.

**Table 5:** Financial losses in milk-equivalent (equivalent to liters of milk), considering the predicted bulk tank somatic cell count (BTSCC) values obtained from time series analysis for each PPBMQ class considering the months of 2016

	Payment per liter of milk in milk-equivalent^a^				
Month	Class 1^1^	Class 2^1^	Class 3^1^	Class 4^1^	Class 5^1^
January/2016	−0.015	−0.015	−0.037	−0.037	−0.045
February/2016	−0.015	−0.015	−0.037	−0.037	−0.045
March/2016	−0.015	−0.015	−0.037	−0.037	−0.045
April/2016	−0.015	−0.015	−0.037	−0.037	−0.045
May/2016	0	−0.015	−0.037	−0.037	−0.045
June/2016	0	−0.015	−0.037	−0.037	−0.045
July/2016	−0.015	−0.015	−0.037	−0.037	−0.045
August/2016	0	−0.015	−0.037	−0.037	−0.045
September/2016	0	−0.015	−0.037	−0.037	−0.045
October/2016	−0.015	−0.015	−0.037	−0.037	−0.045
November/2016	−0.015	−0.015	−0.037	−0.037	−0.045
December/2016	−0.015	−0.030	−0.037	−0.045	−0.045
Annual Average	−0.010	−0.016	−0.037	−0.038	−0.045

^a^Values calculated based in the PPBMQ in Table 1; ^1^Class 1–1000 to 200,000 cells/mL, Class 2–201,000 to 400,000 cells/mL, Class 3–401,000 to 500,000 cells/mL, Class 4–501,000 to 800,000 cells/mL, Class 5 - > 800,000 cells/mL

**Fig. 4:**
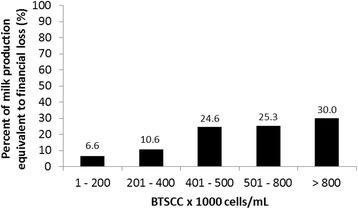
Percent of the milk production equivalent to financial loss based on payment by milk quality. A milk price of R$ 1.34 (1 milk-equivalent) and a profit margin of 15% were used. The payment program based on milk quality (PPBMQ) classes include: Class 1–1000 to 200,000 cells/mL, Class 2–201,000 to 400,000 cells/mL, Class 3–401,000 to 500,000 cells/mL, Class 4–501,000 to 800,000 cells/mL, Class 5 - > 800,000 cells/mL)

The financial loss was calculated considering the price difference between the current class and the best class (class 1). For example, in class 5, the difference was −0.045 milk-equivalents; however, the average value of financial loss was obtained from the time series analysis, and considering that in all the months the future values of BTSCC for such class remained within the class (i.e., with all values >800.000 cells/mL and not changing to other classes – Table 4), the average financial loss for this class was −0.045 milk-equivalents (Table 5). Thus, the milk production equivalent to the financial losses increased with an increase in the PPBMQ class, yielding values of 6.6%, 10.6%, 24.6%, 25.3% and 30.0% for classes 1, 2, 3, 4 and 5 (Fig. 4). These values also represent the number (in %) of lactating cows necessary to achieve such production, based on the average production of the herd in liters/cow/day.

### Applicability: example for a dairy farm

To provide a concrete example, the use of the information contained in this study was simulated for a fictitious farm. The herd had 150 lactating cows with an average production of 25 l/cow/day, equivalent to 3750 l/day. The average BTSCC in the current month was 350,000 cells/mL, and that in the previous month was 315,000 cells/mL. Thus, the difference between the current month and the previous month was 35,000 cells/mL. This difference was divided by the constant *d*_*2*_ (1.128) so the standard deviation for the moving averages could be estimated. In this case, the standard deviation was 31,028 cells/mL.

This farm falls into class 2 (201,000–400,000 cells/mL) for payment based on milk quality. Eq. (5) is the correct one for calculating the class change probability of moving to a worse class. The BTSCC average (350,000 cells/mL) and estimated standard deviation (31,028 cells/mL) are the variables necessary for using eq. (5) to calculate the probability of class change. Solving the equation yields the probability of moving from class 2 to worse classes, which in this case was 31%.

A farm in class 2 loses an average of 0.016 milk-equivalents according to the prediction modeling in this study (Table 5). Considering a profit margin of 15%, a milk price of R$ 1.34 (1 milk-equivalent) and a production of 3750 L/day, eq. (3) indicates that it would be necessary to produce an extra 400 L/day to offset the financial loss. Multiplying 400 L/day for the loss of class 2 (0.016 milk-equivalents) for 30 days, a monthly loss of 12,000 milk-equivalents is obtained. Subsequently, the monthly loss of 12,000 milk-equivalents is multiplied by the current milk price (R$ 1.34) to yield a monthly loss of R$ 16,080.00 due to PPBMQ. This calculation can be applied to other currencies and countries by multiplying the milk-equivalents by the current milk price in that country.

In addition, considering the number of cows in the herd and the average production in liters/cow, the daily milk production necessary to compensate for the financial loss would be approximately 10.6%. Thus, given the current average milk production of the herd, producing the extra 400 l of milk per day would require an additional 16 cows. Finally, it is evident that the farm loses 12,000 milk-equivalents (R$ 16,080.00) per month because the farm falls into class 2 for payment based on milk quality, and the farm has a 31% chance of increasing these losses in the following month if it moves to a worse PPBMQ class.

## Discussion

The results found for the BTSCC during the study period were similar to those found by Machado and Cassoli [9], and it was notable that no recent improvements in terms of the BTSCC were evident. In this study, the increase in the geometric mean of the BTSCC can be partly explained by the increase in the number of farms that have begun to conduct milk analyses over the years (Fig. 1).

It is possible that farms have only recently started to send poor-quality samples, i.e., with higher BTSCC values. However, the increase in the number of farms may be due to the demand for such analyses by the milk buyers, or it may indicate that more farmers are concerned about milk quality and have taken this initiative to analyze the milk produced for this reason.

The probability of passing to a worse class was affected by the BTSCC mean and standard deviation for classes 1 and 2, i.e., the larger the BTSCC mean and the BTSCC variation are in these classes, the greater is the chance that a farm will pass to a worse class in the following month. However, for classes 3 and 4, only the BTSCC mean has such an effect. Therefore, this indicates that BTSCC standard deviation is more influential in the probability of passing to a worse payment class in classes 1 and 2. Thus, the same BTSCC standard deviation is more influential in the probability of passing to a worse payment class in class 1 than in class 3. For example, considering two farms with the same BTSCC standard deviation, but the first in class 1 and the second in class 3, the BTSCC standard deviation will be more influential in the probability of passing to a worse class for the first farm (in class 1) than for the second farm (in class 3). A decrease in the variation of BTSCC is important for process control and for the farmers to reach and remains in the better payment classes [34], as classes 1 and 2.

Using the equations obtained from the multiple linear regression models with eqs. 4, 5, 6 and 7, the probability values for any farm in these classes can be calculated once the BTSCC mean values for two consecutive months are available, and the estimated standard deviation can be obtained as outlined in the methodology of this study. These results can be used as a decision-making method for farmers, technicians and other people in the dairy field. According to Devitt et al. [35], many practices for the improvement of herd health and milk quality developed by researchers in the field may be difficult for dairy farmers to employ, mainly due to a lack of collective action by the government, the dairy industry and experts in the field.

If the class change probability is high, action can be taken to prevent the farm from moving into a class where the price paid per liter of milk is lower or the penalty is higher. In this way, the producer can avoid the financial loss that would accrue with a class change due to payment based on milk quality. Therefore, PPBMQs are important for motivating farmers to make an effort to reduce BTSCC and to maintain it at lower values [2] in order to reduce the variability of the results.

In addition to calculating the probability of passing to a worse class, a correctly adjusted time series model can serve as a decision-making method because it allows a future analysis of the BTSCC results in the different payment classes according to milk quality. If the predicted value and the prediction error indicate that the class limit may be exceeded, action can be taken to prevent the farm from moving to a worse payment class because of poorer milk quality, so the farm does not receive a lower bonus or accrue a penalty due to a class change, considering that farmers with worse results in terms of BTSCC can have stronger intentions to improve than the others [36]. In addition, because of the seasonality of BTSCC, Roma Júnior et al. [7] commented that the seasonal effect on the BTSCC should be considered in the formulation of PPBMQs.

The time series model also allowed us to estimate the average annual financial loss per liter of milk in milk-equivalents for each quality payment class (Table 5). The financial losses for each class were calculated, and it was evident that how much worse the quality payment class, the greater the financial loss and, consequently, the higher the daily milk production that would be required to offset the loss.

Such information could serve as to trigger a farmer to take action to improve milk quality [37] because even those subject to payment based on milk quality based on the BTSCC in Brazil showed poorer rather than improved milk quality as indicated by the BTSCC [9]. It is possible that milk farmers do not realize the importance of the payment based on milk quality (the importance of every cent lost per liter [38]). In this context, all production factor expenditures have already been paid, and any additional revenue is considered a profit [11], which could represent the difference between the success and the failure of the business.

However, even farmers who are in these programs may not feel the need for changes to reduce the BTSCC because of how the programs are executed. Huijps et al. [3] and Valeeva, Lam and Hogeveen [4] have commented that PPBMQs based on penalties are the most effective because of loss aversion, which indicates that people believe that losses are more important than gains [3]. However, none of the tables from milk buyers used in this study to produce the reference table (Table 1) considered a system based exclusively on penalization but used bonus systems only or jointly with a penalty system.

It is possible that the manner in which the tables are presented does not effectively show the farmer the real effect of not achieving the maximum bonus/minimum penalty. According to Bates and Pattisson [39], farmers are informed about milk pricing issues, and they consider this useful, suggesting that the information provided may not be ideal or sufficient to prompt them to improve the BTSCC. Consequently, improving the milk quality will result in a financial benefit, improving the profitability of the farm due to the payment based on milk quality [13]. Therefore, we recommend that the tables be reformulated based on a penalization system exclusively, which has been reported to be more effective.

### Limitations

Only the BTSCC was used as the response variable for the group of farms in this study because other information (fat, protein, total bacterial count, lactose) was available only for a few farms, which limited the data. Therefore, we chose to study the BTSCC to keep the sample *n* representative. Different payment classes based on milk quality can be found among milk buyers and, eventually, cannot be applied to some farms, but our model represents the general situation in Brazil. Because of this, the calculations for the class change probability were restricted to the classes used in the study.

Considering the predicted BTSCC, updated values will be needed in the future for the results to remain valid. Further, additional response variables, such as fat, protein and total bacterial count, can also be predicted in a PPBMQ. Within the time series models, multivariate techniques, such as those proposed by Tsay [17], have emerged with great potential and applicability for dairy science, mainly for indicators and measurements made over time.

## Conclusion

The BTSCC for Brazilian dairy farms has not recently improved. The probability of a change to a worse class is a method that can aid in decision-making and stimulate the farmer to improve milk quality. A time series model can be used to predict the future value of the BTSCC, which makes it possible to estimate financial losses and to show that financial losses occur in all of the PPBMQ classes. This is because farmers do not often remain in the best payment class in all months of the year. Therefore, it is recommended that milk buyers present such information in booklets or payment notes intended for their customers in order to sensitize them to the financial losses due to payments based on milk quality.

## Abbreviations

ACF: Autocorrelation function
AFLC: Average financial losses of the bulk tank somatic cell count class by liter of milk related to the non-attainment of the maximum bonus
AIC: Akaike information criterion
AR: Autoregressive operator
BIC: Bayesian information criterion
BTSCC: Bulk tank somatic cell count
CEPEA: Center for Advanced Studies in Applied Economics
DP: Daily production in liters of milk
ESALQ: College of Agriculture “Luiz de Queiroz”
MA: Moving-averages operator
MP: Milk price
PACF: Partial autocorrelation function
PM: Profit margin
PPBMQ: Payment program based on milk quality
PROB: Class change probability
SARIMA: Seasonal autoregressive integrated moving average model
SCC: Somatic cell count
STD: Estimated bulk tank somatic cell count standard deviation of the farm
TBC: Total bacterial count
USP: University of São Paulo
